# Evaluation of self-perception of mechanical ventilation knowledge among Brazilian final-year medical students, residents and emergency physicians

**DOI:** 10.6061/clinics/2017(02)01

**Published:** 2017-02

**Authors:** Fernando Sabia Tallo, Simone de Campos Vieira Abib, Alexandre Jorgi de Andrade Negri, Paulo Cesar Filho, Renato Delascio Lopes, Antônio Carlos Lopes

**Affiliations:** I Universidade Federal de São Paulo (UNIFESP), Departamento de Cirurgia, São Paulo/SP, Brazil; II Universidade Federal da Paraíba, Departamento de Cardiologia, João Pessoa/PB, Brazil; III Universidade Evangélica de Anapolis, Anapolis/GO, Brazil; IV Duke University Medical Center, Duke Clinical Research Institute, Durham, North Carolina, United States

**Keywords:** Artificial Respiration, Medical Education, Emergency Medicine

## Abstract

**OBJECTIVE:**

To present self-assessments of knowledge about mechanical ventilation made by final-year medical students, residents, and physicians taking qualifying courses at the Brazilian Society of Internal Medicine who work in urgent and emergency settings.

**METHODS:**

A 34-item questionnaire comprising different areas of knowledge and training in mechanical ventilation was given to 806 medical students, residents, and participants in qualifying courses at 11 medical schools in Brazil. The questionnaire’s self-assessment items for knowledge were transformed into scores.

**RESULTS:**

The average score among all participants was 21% (0-100%). Of the total, 85% respondents felt they did not receive sufficient information about mechanical ventilation during medical training. Additionally, 77% of the group reported that they would not know when to start noninvasive ventilation in a patient, and 81%, 81%, and 89% would not know how to start volume control, pressure control and pressure support ventilation modes, respectively. Furthermore, 86.4% and 94% of the participants believed they would not identify the basic principles of mechanical ventilation in patients with obstructive pulmonary disease and acute respiratory distress syndrome, respectively, and would feel insecure beginning ventilation. Finally, 77% said they would fear for the safety of a patient requiring invasive mechanical ventilation under their care.

**CONCLUSION:**

Self-assessment of knowledge and self-perception of safety for managing mechanical ventilation were deficient among residents, students and emergency physicians from a sample in Brazil.

## INTRODUCTION

Mechanical ventilation is an expensive intervention with high morbidity and mortality, and the complexity of the procedure requires a specially trained working team 1,2. The manner in which mechanical ventilation is applied affects both primary and secondary patient outcomes 3-5, and best practices are not consistently applied 6. This inadequacy is partially due to the lack of proper training received by physicians in mechanical ventilation 7.

Even when inadequate mechanical ventilation practices are used for only brief periods, hospital stays are often prolonged, and patients can experience mechanical ventilation-induced injuries 8-10. These consequences are becoming increasingly common across various hospital departments 11,12. Exacerbating the situation, many physicians with no formal training in mechanical ventilation end up managing critically ill patients undergoing this intervention 13-16.

We conducted a cross-sectional, descriptive study wherein final-year medical students, residents and physicians who work in emergency rooms in Brazil were asked to assess their knowledge about mechanical ventilation.

## METHODS

Following approval by the ethics and research committee of Universidade Federal de São Paulo (UNIFESP), an observational, cross-sectional, descriptive study was conducted in which final-year medical students, residents, and emergency physicians were asked to assess their knowledge about mechanical ventilation through a 34-question semi-structured questionnaire (Appendix 1).

In the first phase, a committee was formed by the main investigator, four physicians who specialize in adult intensive care, a pulmonologist, and a professor of graduate and post-graduate courses at the medical school of UNIFESP to create the questionnaire. Standardization was achieved using Delphi’s technique, which was used to define the content needed to describe competent practice of mechanical ventilation. This version of the questionnaire contained 56 items on the following topics: learning objectives 17, respiratory physiology (mechanics), ventilation modes, patient-ventilator interactions, noninvasive ventilation, mechanical ventilation in (COPD) and (ARDS), and mechanical ventilation weaning.

In the second phase, a pilot project was carried out with sixth-year students from UNIFESP and intensive care senior physicians. Following this, adjustments in the wording of the questions were made, and certain items were excluded or included based on observations from the first phase. The final revision of the questionnaire contained 34 items. The third phase of the study occurred from from August 2013 to July 2015 and focused on data collection. During this phase, questionnaires and informed consent forms were sent to students and coordinators of 24 institutions. Individuals from the following eleven institutions agreed to participate: School of Medicine of the City of Fernandópolis, School of Medical Sciences of the State of Minas Gerais, School of Medicine of Unichristus, Federal University of the State of Paraíba, School of Medicine of UNICEUMA, Santa Casa de Ribeirão Preto, Federal University of the State of Píaui, School of Medicine of the City of Pouso Alegre, School of Medicine of the City of Blumenau, Unievangélica of the City of Anapolis, Federal University of the State of Minas Gerais and Federal University of the State of São Paulo (UNIFESP). Other participants in the study were emergency physicians enrolled in courses at the Brazilian Society of Internal Medicine and the Brazilian Association of Urgency and Emergency: VMURGEM (Mechanical ventilation in emergency services), located in the cities of Fortaleza, Juazeiro do Norte, Belo Horizonte, Juiz de Fora, Porto Alegre, São Paulo, Camboriú and Teresina. These participants were physicians working in emergency services who were not residents or in a residency program, were not students and did not work in the ICU. The distribution of the participants by employment is shown in Table 1.

The questionnaires were distributed to 1251 individuals only after the coordinators of the various programs confirmed their interest in participating. The questionnaires were considered complete when more than 32 questions were answered. All physicians selected from the training courses agreed to participate in the survey directly through the main investigator. The remaining questionnaires were distributed only after agreement to participate by the program coordinator.

After the participants assessed their knowledge, scores were created based on their answers. All answers that were considered positive according to self-perceived knowledge or that had positively contributed to self-perceived knowledge were summed and converted into a percentage with the same individual weight (0-100%).

The study used SPSS (Statistical Package for Social Sciences), version 21.0 for data analysis. The responses of all participants for each item were described. Scores among the categories were compared using the Kruskal-Wallis nonparametric statistical test. Spearman’s correlation analysis was used to correlate the year of medical residency with the questionnaire score.

## RESULTS

A total of 806 questionnaires were completed and included in the study. The respondents included 448 students (56%), 103 first-year residents (13%), 96 second-year residents (12%), 32 third-year residents (4%) and 127 emergency physicians (16%).

**Figure 1:**
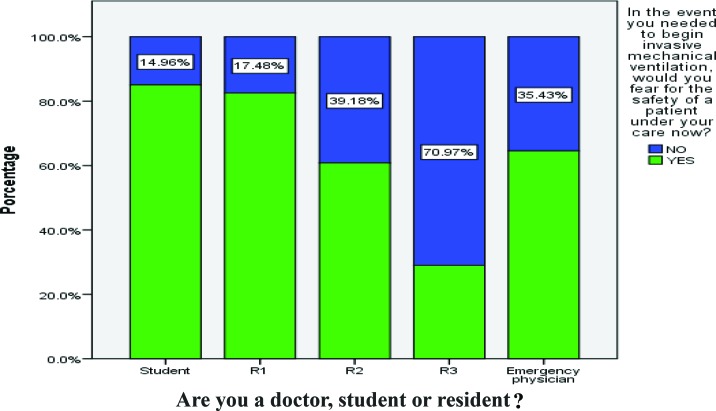
Students, residents and physicians in the study sample.

The mean score across all categories for the physicians who answered the questionnaire was 21% (Table 1). Of all the participants, 720 (89%) felt that they did not receive sufficient information during training to manage a patient on mechanical ventilation. Additionally, 46% felt that there was a lack of professionals available for teaching, 69% (546) said that they would have difficulty turning on a ventilator, and 77% (621) stated they would fear for the safety of a patient if they had to start mechanical ventilation (Figure 1). Regarding the specific skills needed to initiate mechanical ventilation, 77% said that they would not know how to begin non-invasive ventilation in a patient, and 81%, 81% and 89% said they would not be able to start pressure-controlled ventilation (PCV), volume-controlled ventilation (VCV), or pressure support ventilation (PSV), respectively (Tables 2, 3 and 4).

Regarding patient monitoring, 79% did not feel able to deliver adequate sedation to patients, 85% had never used a pain scale, and 89%, and 88% did not feel able to set alarms or to properly react when facing patient-ventilator asynchrony. Additionally, 59% stated they would start ventilation based on a patient's weight in kilograms, and 82% said they would not feel capable of conducting ventilation weaning. Furthermore, 86% of the participants felt incapable of starting mechanical ventilation in patients with chronic obstructive pulmonary disease, and 94% said they would not know how to start ventilation in patients with acute respiratory distress syndrome. Regarding respiratory monitoring, 84%, 93%, 87%, 85% and 92% were not aware of the concepts of resistance, compliance, time constant, plateau pressure and driving pressure, respectively. Additionally, 83% were not aware of the concept of auto-PEEP, and 96% would not know how to estimate it at bedside. The overall mean score of the participants was 21%. Performance by category is shown in Table 1. There was a significant difference between the different groups (*p*=0.0001). Comparing the groups, the poorest performance was found among students (17%). First-year residents and physicians working in emergency services showed similar self-assessment scores of 21% and 22% (*p*=0.52), while second-year residents had a mean score of 31% and third-year residents a mean score of 45%.

The third-year residents felt more secure in their knowledge regarding mechanical ventilation compared with the other groups (R3, 71.0%; students, 15.0%; R2, 36.1%; R1, 16.0%; physicians, 34.6%; *p*<0,0001) (Figure 1).

## DISCUSSION

The present study evaluated the self-assessed knowledge of mechanical ventilation held by various groups of physicians. Few studies have evaluated this topic using validated instruments 18. Studies performed in residency training centers for emergency services have typically revealed that there are no specific educational programs regarding mechanical ventilation 19-21.

In Brazil, there is no emergency medicine specialty, and most physicians working in this area of health are relatively recent graduates (having graduated less than five years before entering the field). As such, they have not received any specific training in mechanical ventilation 22.

The average scores among the different assessed categories were very low. The average overall knowledge score did not exceed 45% in any group. The best scores were found for third-year physicians training in internal medicine, but their average score was still only 45%. The knowledge scores for first-year residents and emergency physicians were similar, at 21% and 22%, respectively (*p*=0.52).

Most students (77%) had never handled an artificial ventilator, and 45% indicated that there was a lack of specialists available to teach this subject in the programs they attended.

Ventilation strategies for setting tidal volume, PEEP, and opening airway pressure and the monitoring of auto-PEEP, plateau pressure, driving pressure and peak pressure can affect mortality 23-27 and cause mechanical ventilation-induced injury 28,29. Only 14.8% of the respondents indicated they knew how to measure plateau pressure at bedside, 93% rated themselves as not knowledgeable of the concept of driving pressure, and 6.3% reported not knowing how to calculate static compliance.

Mechanical ventilation is used to manage patients with chronic obstructive pulmonary disease 22,30, severe acute asthma 31 and acute respiratory distress syndrome 32,33. Most physicians stated they did not know the principles for applying ventilation to these patients: 87% did not know how to manage mechanical ventilation for chronic obstructive pulmonary disease patients, and 94% did not know how to manage it for acute respiratory distress syndrome patients.

Noninvasive mechanical ventilation can reduce the mortality of patients with chronic obstructive pulmonary disease exacerbation and severe respiratory failure 34, acute cardiogenic pulmonary edema 35, and other emergent respiratory acute cases. Despite this, 77.2% of the physicians in training, active physicians, and medical students surveyed in this work did not know how to initiate the intervention.

In critically ill patients on mechanical ventilation, evaluation and treatment with sedation and analgesia should be routinely performed 36. This systematic evaluation of the level of sedation and pain should be performed with the aid of behavioral 37-39 and sedation 40,41 scales adapted and translated into Portuguese. Such monitoring decreases the duration of mechanical ventilation, reduces nosocomial infections, and lessens the incidence of pain and agitation 42. Of all the respondents, 86% had never used a pain assessment scale during training or in their professional work, and 79% did not feel able to administer sedation and analgesia to mechanically ventilated patients.

Delayed weaning from mechanical ventilation lengthens the duration of mechanical ventilation and increases mortality 43,44. Several factors 45 have been reported to increase the success of this process, but 83% of the study participants reported not knowing the principles involved.

Knowledge concerning conventional modes of mechanical ventilation, namely, PCV, VCV, and PSV, was very low in the sample, as was knowledge regarding ventilation strategies for special situations such as in patients with chronic obstructive pulmonary disease or acute respiratory distress syndrome. Of the third-year residents, who had the highest overall scores, 52% expressed that they would be able to ventilate an obstructive patient, whereas only 22% felt able to ventilate a patient with acute respiratory distress syndrome. Increased exposure time to mechanical ventilation during training increased the feeling of confidence. Unfortunately, there is no formal program for teaching this subject in medical school, which might explain the low scores.

In intensive care practice, 70% of adverse events related to mechanical ventilation are related to human errors 46. As there are reports that adverse events are underreported among active physicians and residents in clinical practice 47,48, it is likely that this value is even higher in the field of emergency service. In our sample, 29.9% of respondents had never handled an artificial ventilator, and 60.9% thought they would have difficulty even turning such a device on. Furthermore, 80% did not know what a regulating or reducing valve was, and 89% did not know any technical standards for using medical gases. This implies the need to develop educational programming for risk management among training and active physicians.

### Limitations

One limitation to the present study is the use of a questionnaire for self-assessment of knowledge rather than a direct measure of competence in mechanical ventilation. Furthermore, our use of convenience sampling among participants with heterogeneous characteristics may have influenced our results. It is possible that scores were underestimated due to the greater likelihood of residents, students and physicians with low levels of knowledge regarding mechanical ventilation refusing to participate in the study.

The level of knowledge about various components of mechanical ventilation was low among the participants in the sample. Specifically, there was a low level of knowledge about respiratory mechanics, conventional modes, monitoring, strategies for special situations, and weaning from mechanical ventilation. The participants also felt that teaching about mechanical ventilation in medical school and in medical residency programs was lacking. Additional studies evaluating competence in using mechanical ventilation among training and active physicians are needed. Our results indicate the need for a dedicated medical education program for mechanical ventilation for all medical students and an extended training program for physicians practicing emergency care.

## AUTHOR CONTRIBUTIONS

Tallo FS collected the data, wrote the manuscript and was responsible for statistical analysis. Abib SC conceived and designed the study and was responsible for data interpretation. Negri AJ and Filho PC were responsible for data acquisition. Lopes RD was responsible for data interpretation and statistical analysis. Lopes AC critically revised the manuscript and was responsible for statistical analysis.

## APPENDIX

### QUESTIONNAIRE FOR SELF-ASSESSMENT OF KNOWLEDGE ABOUT MECHANICAL VENTILATION

### OBJECTIVE

**To assess the level of knowledge about mechanical ventilation held by students who completed medical training in Brazil as well as resident physicians in training for internal medicine and physicians working in emergency services.**

Age ______ Sex: male ( ) female ( )

Resident ( ) year ________ Student ( ) year __________

Resident physician ( ) year of graduation ____________

School

**Table:** 

**Choose the answer for the questions below according to what is stated.**	
1. Do you believe that during your medical training you received sufficient information to manage the mechanical ventilation patient?	Yes ( ) No ( )
2. Have you ever handled an artificial ventilator during the course of your training or in your medical residency program?	Yes ( ) No ( )
3. Is it reasonable to say that you would have difficulties even turning a mechanical ventilation device on?	Yes ( ) No ( )
4. Would you fear for the safety of a patient you are caring for if invasive mechanical ventilation was required?	Yes ( ) No ( )
5. Do you currently have a patient in the emergency room requiring noninvasive mechanical ventilation? Would you be able to begin this intervention for this patient?	Yes ( ) No ( )
6. Would you be able to safely set a ventilator to controlled pressure mode?	Yes ( ) No ( )
7. Would you be able to safely set a ventilator to controlled volume mode?	Yes ( ) No ( )
8. Would you be able to set a ventilator to pressure support mode?	Yes ( ) No ( )
9. Do you know what patient-ventilator interactions can occur and their possible clinical implications?	Yes ( ) No ( )
10. In your medical school or residency program, do you believe there is a sufficient number of teaching physicians who are able to guide you on mechanical ventilation?	Yes ( ) No ( )
11. Do you think you could establish adequate sedation in a patient on mechanical ventilation?	Yes ( ) No ( )
12. I am dissatisfied with my knowledge about mechanical ventilation, and I think I should improve it.	Yes ( ) No ( )
13. Do you think that a physician should bypass gaining knowledge about mechanical ventilation because another professional will be able to help?	Yes ( ) No ( )
14. You are treating a 30-year-old healthy patient weighing 120 kg measured on a perfectly calibrated scale. There is indication for invasive mechanical ventilation that is not related to respiratory or cardiovascular medical conditions. Suppose you have to set a tidal volume. What is your suggestion for ventilation in relation only to the tidal volume provided?	a) Only 4 ml/kg of body weight.b) 10 ml/kg of body weight because the patient is healthy.c) To be sincere, I would not know. I would ask another professional.d) There are not enough elements to answer this question.
15. You need to start mechanical ventilation in a patient using an oxygen torpedo tube. The nurse provides a valve with a flow meter to be used. It is noted that the device does not “cycle”, although the torpedo is full, and the valve and machine are in perfect working order. Would you know what could be happening?	Yes ( ) No ( )
16. Do you know any technical standard associated with the use of medical gases?	Yes ( ) No ( )
17. Have you ever used a specific scale of pain evaluation in patients on mechanical ventilation? Choose true or false.	Yes ( ) No ( )
18. I know how to set the alarms according to the clinical situation.	T ( ) F ( )
19. The disconnection alarm should always be set by the operator.	T ( ) F ( )
20. I know how to identify the main graphs of mechanical signs in mechanical ventilation.	T ( ) F ( )
21. I know the concept of resistance of the respiratory system, and I know how to identify possible changes from observing the graphs.	T ( ) F ( )
22. I know the concept of respiratory system compliance and how to identify possible changes from observing the graphs.	T ( ) F ( )
23. I can calculate respiratory system static compliance.	T ( ) F ( )
24. I can calculate respiratory system dynamic compliance.	T ( ) F ( )
25. I know the concept of time constant.	T ( ) F ( )
26. I know the basic principles of COPD patient ventilation, and I am confident I could initiate them if required.	T ( ) F ( )
27. I know the evidence-based principles of mechanical ventilation for ARDS, and I am confident that I could initiate them if required.	T ( ) F ( )
28. I know the principles for using PEEP, and I am confident that I can set it if required.	T ( ) F ( )
29. I know the prophylaxis measures for mechanical ventilation-associated pneumonia.	T ( ) F ( )
30. I know how to measure the plateau pressure at bedside, I know the recommended limits, and I know the possible impacts on mechanical ventilation.	T ( ) F ( )
31. I know the concept of auto-PEEP and its implications.	T ( ) F ( )
32. I know how to measure auto–PEEP at bedside.	T ( ) F ( )
33. I know the concept of driving pressure.	T ( ) F ( )
34. I know the principles of weaning from mechanical ventilation.	T ( ) F ( )

## Figures and Tables

**Table 1:** Scores for the students, internal medicine residents, and physicians in the study sample.

Category	Score	N
Students	17%	448
R1	21%	103
R2	31%	97
R3	45%	31
Emergency physicians	22%	127

R1: first-year residents, R2: second-year residents, R3: third-year residents. Scores ranged from 0-100%. The students were in the sixth year of medical school.

**Table 2:** Percentage of physicians who considered themselves able to set the PCV mode.

Category	Yes	%
Students	45	10%
R1	26	25%
R2	25	25%
R3	21	68%
Emergency physicians	39	34%
Total	156	19%

**Table 3:** Percentage of physicians who considered themselves able to set the VCV mode.

Category	YES	%
Students	45	10%
R1	26	25%
R2	22	25%
R3	23	74%
Emergency physicians	40	31%
Total	156	19%

**Table 4:** Percentage of physicians who considered themselves able to set the PSV mode.

Category	Yes	%
Students	22	5%
R1	16	15%
R2	22	23%
R3	7	22%
Emergency physicians	24	19%
Total	91	100%
